# Trends in global research on tobacco use among sexual and gender minorities: A bibliometric analysis, 1984–2024

**DOI:** 10.18332/tid/208740

**Published:** 2025-10-07

**Authors:** Lei Qiu, Zhang Shirui, Muyuan Luo

**Affiliations:** 1 School of Foreign Languages, Huaiyin Normal University, Huai’an, China; 2 Research Institute of Social Development, Southwestern University of Finance and Economics, Chengdu, China

**Keywords:** sexual and gender minorities (SGM), tobacco use, bibliometric analysis

## Abstract

**INTRODUCTION:**

Globally, sexual and gender minorities (SGM) exhibit significantly higher tobacco use rates than their cisgender heterosexual counterparts, a persistent health disparity that has garnered increasing attention in public health research.

**METHODS:**

We conducted a bibliometric analysis of 704 SGM tobacco use-related publications from the Web of Science Core Collection. First, we examined publication trends, key contributors, and collaborative networks. Second, we performed co-citation network analysis to identify disciplinary characteristics and research hotspots. Finally, we applied keyword burst detection and clustering techniques to assess emerging trends and frontier areas.

**RESULTS:**

From 1984 to 2024, research on SGM tobacco use demonstrated consistent growth. The US accounted for the majority of publications (82.52%), with institutions such as the University of California System serving as key hubs for research collaboration. Research hotspots clustered around five key themes: 1) the effects of novel tobacco products, 2) subgroup differences in tobacco use, 3) tobacco-related health disparities, 4) smoking cessation research, and 5) social and psychological mechanisms.

**CONCLUSIONS:**

Using data mining and visualization techniques, this study constructed a comprehensive knowledge map of research on SGM tobacco use. Our findings elucidate evolving patterns and emerging trends while offering valuable perspectives to guide future investigations.

## INTRODUCTION

Although global smoking prevalence has declined in recent years^1^, tobacco use among sexual and gender minorities (SGM) remains a pressing public health concern. Extensive empirical evidence indicates that SGM adolescents and adults use tobacco at significantly higher rates than their cisgender heterosexual counterparts, whose gender identity aligns with their sex assigned at birth^2,3^. For instance, in the US, LGBT adults exhibit a 68% higher smoking prevalence than cisgender heterosexual adults^4^. Notably, SGM women – including cisgender and transgender lesbian and bisexual individuals – are twice as likely to smoke as cisgender heterosexual women^5^. Although global data remain limited, population-based studies in other countries confirm similar disparities^6^. Moreover, emerging tobacco products, particularly e-cigarettes, are increasingly used by SGM populations^7^. Notably, transgender adults use e-cigarettes at more than four times the rate of cisgender adults^8^. This disproportionate e-cigarette use contributes significantly to rising tobacco consumption in SGM communities.

Consequently, SGM individuals face elevated risks of tobacco-related health complications^9^. SGM smokers exhibit heightened risks of cardiovascular disease, HIV, asthma, cancer, and hepatitis^10^. Furthermore, tobacco use exacerbates age-related chronic disease risks among older SGM adults, severely compromising their quality of life^11^. This disparity stems from multiple factors, including minority stress, social normalization of tobacco use, and targeted marketing^12^. Marginalization-related stressors uniquely experienced by SGM individuals further drive tobacco use^13^. Additionally, pervasive tobacco advertising disproportionately targets SGM communities, reinforcing higher usage rates^14^. Moreover, SGM individuals’ heightened sensitivity to environmental influences during identity development compounds barriers to effective tobacco control policies and cessation services^15^.

In recent years, public health researchers, tobacco control experts, and SGM advocates have increasingly prioritized addressing these disparities^16^. Understanding the root causes of these disparities is critical for developing evidence-based interventions and policies – an urgent priority in public health research. However, a comprehensive synthesis of global research on SGM tobacco use remains lacking. Bibliometric analysis offers an objective, systematic approach to map research trends, collaborations, and gaps in studies on SGM tobacco use. Unlike traditional qualitative reviews, which may overlook key literature, bibliometrics quantifies and visualizes large datasets to uncover thematic evolution, research clusters, and under-explored gaps. This study employs bibliometric methods to address three key questions: 1) ‘What are the dominant patterns of knowledge production in research on SGM tobacco use?’; 2) ‘How have collaborative and interdisciplinary networks evolved?’; and 3) ‘What are the current research hotspots and emerging trends?’.

## METHODS

### Data collection

This study focuses on SGM and tobacco use and was searched from the WoS Core Collection. WoS is known for its systematic and rigorous selection criteria, authoritative data, stable information, and wide range of resources, making it one of the best sources of data for bibliometric analyses^17^. The search string included: TS=[(Sexual-minorit* OR Gender-Minorit* OR gender-expansive OR gender-diverse OR gender-identit* OR sexual-identit* OR gender-nonconforming OR gender-dysphoria OR sex-attract* OR same-sex OR gender-variant OR sexual-orientation OR LGB OR LBG OR GLB OR LGBT OR LGTB OR LBGT OR LGBTQ OR LGBTQQ OR LGBTTQ OR LGBTQ2 OR LGBTQ2S OR LGBTQ2SIA OR LGBTQIA OR LGBTQAI OR LGBTQA OR LGBTQI OR LGBTQIA2S OR LGBTI OR LGBTIQ OR LGBTIQA OR GBLT OR GLBT OR GLBTI OR GLBTQ OR BGLT OR TGNC OR Lesbian OR gay OR Bisexual* OR transgender* OR queer OR pansexual OR intersex OR asexual OR transsexual* OR homosexual* OR non-heterosexual* OR Men-who-have-sex-with-men OR women-who-have-sex-with-women OR two-spirit) AND (cigarette OR tobacco OR smok* OR nicotine)].

Second, the search results were further qualified: document type = article + review; and language = English. The final search, conducted on 31 December 2024, yielded 2904 initial records. Two researchers independently screened the search results to exclude duplicate records and articles whose titles, keywords, and abstracts did not contain references to tobacco use or SGM. Discrepancies were resolved through consensus discussions. After this process, 704 articles met the inclusion criteria for analysis.

### Analytical methods

In line with EQUATOR Network guidance, this bibliometric analysis followed the recommended reporting standards for bibliometric studies. This study employed bibliometric methods using CiteSpace (6.2.R3) to analyze the knowledge structure and evolutionary trends in research on SGM tobacco use, a bibliometric software tool that has gained widespread acceptance in academic circles. First, we conducted performance analysis to characterize the dynamic development of research outputs in this field, through which we identified the ten most academically influential core journals. Additionally, we identified the most active authors by applying Price’s law $\left(M=0.749\sqrt{N_{\max}}\right)$, a core author formula that determines productivity thresholds for leading researchers in the field^18^. Second, we constructed a comprehensive research collaboration network through analysis of four key dimensions: transnational cooperation, institutional collaboration, author co-occurrence, and disciplinary crossover. For network analysis, we employed betweenness centrality (BC) to evaluate node importance. This metric quantifies a node’s hub status by measuring how frequently it serves as the shortest path intermediary between other node pairs, thereby indicating its control over information flow and resources within the network. We also examined co-citation patterns, which occur when two documents are cited simultaneously by a third document. These patterns reveal meaningful associations and evolutionary trajectories within the discipline’s knowledge structure^19^. Finally, the research hotspots and their evolutionary trajectories are systematically identified through keyword burst detection and cluster analysis, in combination with time-series slicing. Keyword burst detection quantifies significant changes in specific keywords over short time periods, aiming to identify research topics that have rapidly gained academic attention. This technique is widely used for dynamic monitoring of cutting-edge fields and recognizing emerging research frontiers. Meanwhile, spectral clustering is employed to intelligently group literature data. By extracting representative keywords from each cluster, a research frontier map with clear semantic direction is generated, which reveals thematic relationships among various research hotspots and visualizes the boundaries between disciplinary domains^20^. When combined, these methods provide a comprehensive view of knowledge diffusion processes and frontier evolution within the field. The complete analytical workflow is presented in Supplementary file Figure 1.

## RESULTS

### Basic attributes

Supplementary file Figure 2 illustrates the change in publication volume from 1984 to 2024. Overall, the number of publications demonstrates a clear upward trend, with a total of 704 articles published during this period. During the first decade (1984–1993), annual publication output remained low, averaging fewer than 5 articles per year. Research interest grew substantially beginning in 2003, with annual publications reaching 39 by 2014. Following this milestone, publication numbers showed a fluctuating but steady upward trajectory, ultimately reaching 87 publications in 2024. This growth pattern reflects increasing scholarly attention to tobacco use among sexual minorities. Globally, research on tobacco use behaviors among sexual minorities has appeared in 245 distinct journals. As detailed in Supplementary file Table 1, the most prolific journals were Nicotine & Tobacco Research (58 articles), LGBT Health (40 articles), and Drug and Alcohol Dependence (31 articles). Notably, two leading public health journals – Substance Use & Misuse and American Journal of Public Health – tied for fourth position (27 articles each), underscoring the policy relevance of this research area. Furthermore, substance abuse journals collectively accounted for 38% of publications, including Drug and Alcohol Dependence (31 articles) and Addictive Behaviors (24 articles). This pattern indicates that researchers frequently examine tobacco use within the broader context of addictive behaviors in SGM populations.

### Collaboration network and performance analysis

Analysis of research scholars, institutions, and countries provides valuable perspectives into knowledge development trajectories and future research directions. This section examines global research distribution and collaboration patterns through analysis of the top 10 contributing countries, institutions, and authors in research on SGM tobacco use (Table 1). The US dominates research output with 581 publications and demonstrates the highest influence (BC=1.08). Canada ranks second (N=49; BC=0.31), followed by the UK (N=25) and Australia (N=18). Canada serves as the primary international collaborator after the US. Research influence concentrates in Europe and North America, while Asian representation remains limited – China ranks fifth with 15 publications, followed by five other countries with minimal output and centrality. The University of California System leads institutional output (N=74), followed by Harvard University (N=50) and University of California San Francisco (N=40). The University of California System demonstrates both quantitative leadership and strong network centrality (BC=0.24), indicating its key role in institutional collaborations. These institutions, along with Columbia University (N=36, BC=0.08) and the University System of Ohio (N=36, BC=0.19), serve as crucial bridges in global research networks. Notably, all top ten institutions are US-based, predominantly university systems (e.g. University of Texas System, Pennsylvania Commonwealth System). Their high citation rates further underscore US dominance in this field. Between 1986 and 2024, 722 researchers contributed to studies of SGM tobacco use. As shown in Table 1, the most prolific authors are McCabe (N=15), Berg (N=14), and Boyd (N=14). Notably, Lee (N=12) and Matthews (N=10) demonstrate particularly strong bridging roles (BC=0.02 each), despite their slightly lower publication counts. Analysis reveals that highly productive authors (>10 publications) operate in relatively dispersed collaborative networks, as most show BC values of zero. This pattern suggests significant potential for strengthened research collaborations.

**Table 1:** Top 10 most productive countries, institutions and authors, 1984–2024 (N=704)

*Ranking*	*Institution*	*Count*	*BC*	*Country*	*Count*	*BC*	*Author*	*Count*	*BC*
1	University of California System	74	0.24	USA	581	1.08	Mccabe, Sean Esteban	15	0
2	Harvard University	50	0.13	Canada	49	0.13	Berg, Carla J.	14	0
3	University of California San Francisco	40	0.09	England	25	0.12	Boyd, Carol J.	14	0
4	University of Texas System	38	0.02	Australia	18	0.01	Lee, Joseph G. L.	12	0.02
5	Columbia University	36	0.08	China	13	0	Azagba, Sunday	11	0
6	University System of Ohio	36	0.19	Brazil	9	0.05	Matthews, Alicia K.	10	0.02
7	University of Michigan	36	0.02	Mexico	9	0	Evans-Polce, Rebecca J.	10	0
8	University of Michigan System	36	0.02	India	8	0	Hughes, Tonda L.	10	0
9	Harvard Medical School	31	0.02	Netherlands	8	0.01	Fish, Jessica N.	10	0.01
10	Pennsylvania Commonwealth System of Higher Education (PCSHE)	30	0.2	Italy	7	0.05	Watson, Ryan J.	10	0

BC: betweenness centrality.

### Disciplinary development and collaboration

**Figure 1:**
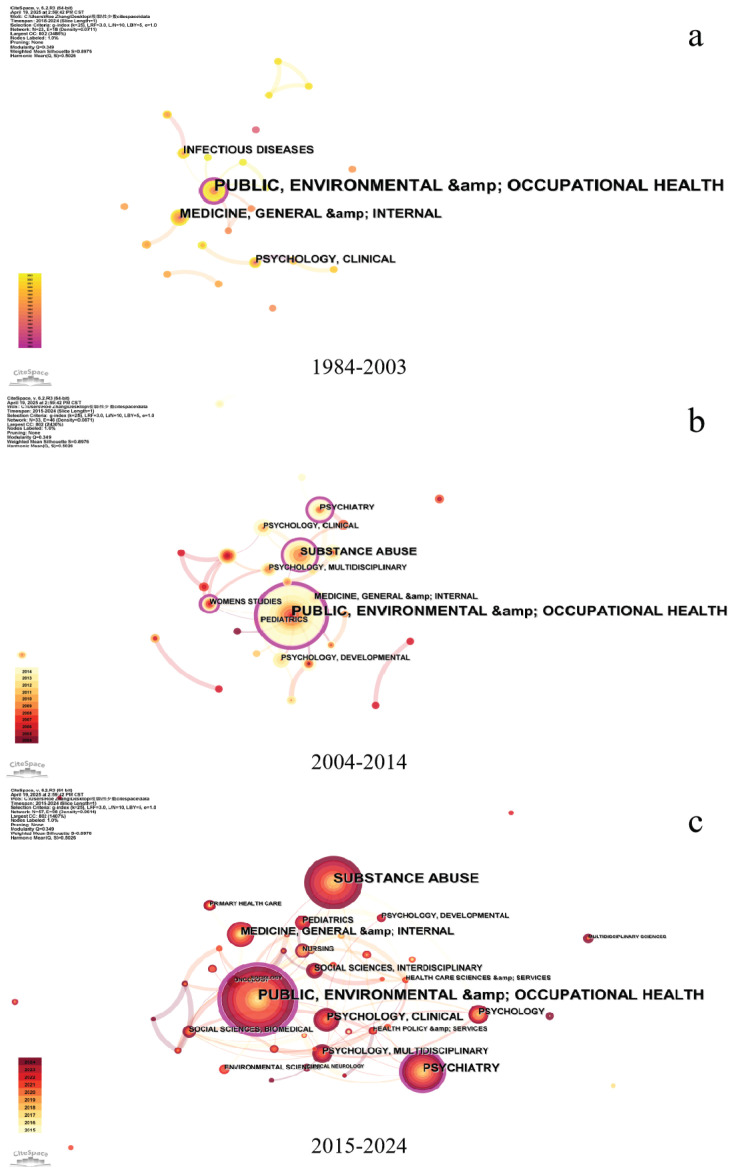
Category collaboration networks for 1984–2003, 2004–2014, and 2015–2024. Each node indicates a category, and the larger the node, the more articles were published. Each edge indicates a collaborative relationship between two categories

Figure 1 demonstrates the disciplinary evolution and expansion within global research on minority tobacco use. Analysis of historical publication data reveals three distinct developmental phases: 1) 1984–2003, 2) 2004–2014, and 3) 2015–2024. The initial phase (1984–2003) was characterized by research predominantly in public health and medicine. During the second phase (2004–2014), research interest grew substantially, expanding the disciplinary scope to establish public health, sociology, and psychology as core research domains. The contemporary phase (2015–2024) has been marked by two significant developments: 1) the globalization and diversification of tobacco consumption patterns among minority populations; and 2) the emergence of a robust interdisciplinary research paradigm encompassing epidemiology, health policy, social work, pedagogical studies, and cultural anthropology. This scholarly convergence has substantially enriched both the methodological diversity and theoretical depth of academic inquiry in this domain.

### Research hotspots and trends

**Figure 2:**
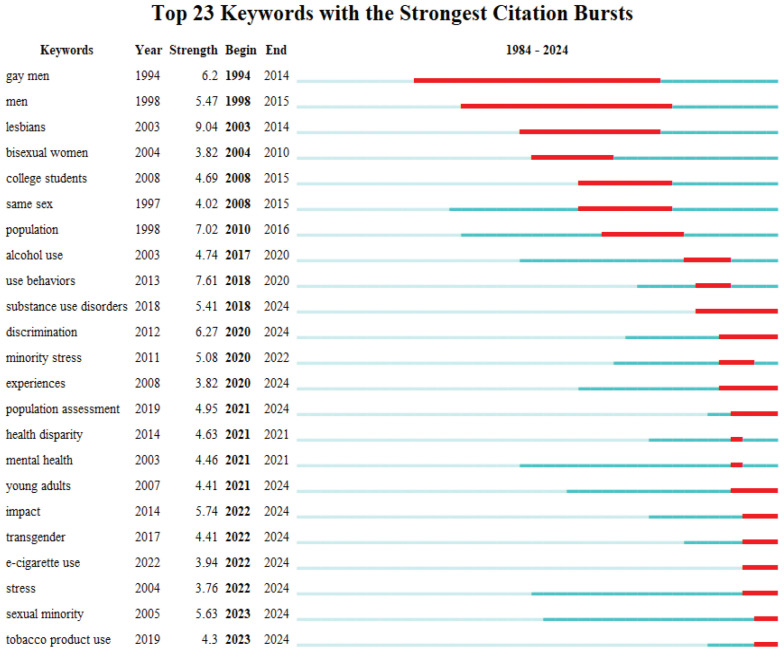
The keywords with the strongest citation bursts. The beginning of a blue line depicts when an article is published. The beginning of a red segment marks the beginning of a period of burst, whereas the end of the red segment marks the end of the burst period or the end of the observation window (1986–2024) (N=704)

To reveal the evolutionary trajectory of global research on tobacco use among minority populations, we conducted a keyword burst detection based on emergence intensities from 1984 to 2024 (Figure 2), with the burst detection threshold set at 0.7. In the early phase (1994–2015), research exhibited distinct population specificity, with terms like ‘gay men’ (intensity = 6.2) and ‘lesbians’ (intensity = 9.04) emerging prominently. However, the burst cycles of these descriptive keywords largely concluded before 2015. During the 2017–2020 period, the continued prominence of keywords such as substance use disorders’ (intensity = 5.41) and ‘alcohol use’ (intensity = 4.74) suggests an academic shift toward investigating co-morbid patterns between tobacco use and other substances. Over the past five years (2020–2024), three major research frontiers have emerged. First, structural discrimination factors have gained increased attention. The strong emergence of ‘discrimination’ (intensity = 6.27) and ‘minority stress’ (intensity = 5.08) indicates that current scholarship is increasingly focused on the underlying causes of smoking among sexual minorities. Second, the rise of emerging tobacco products has become a focal point of recent research. Terms such as ‘e-cigarette use’ (intensity = 3.94) and ‘tobacco product use’ (intensity = 4.30) have surged in parallel with the development of novel tobacco technologies. Finally, terms like ‘sexual minority’ (intensity = 5.63) and ‘health disparity’ (intensity = 4.63) have continued to emerge through 2024. Notably, the recent emergence of the term ‘transgender’ (2022–2024), in close temporal alignment with ‘mental health’ (r=0.82, p<0.01), strongly suggests that addressing tobacco use among sexual minorities requires psychological-level interventions.

### Timeline view of keyword co-citation clusters

**Figure 3:**
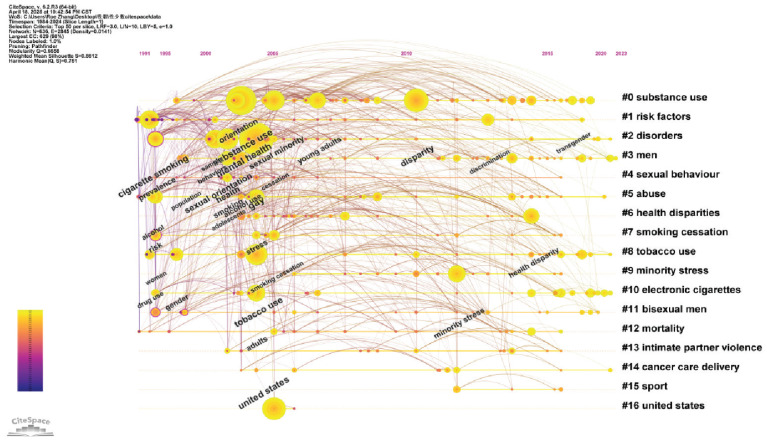
Timeline view of keyword co-citation clusters. The clustering quality is considered satisfactory, with a modularity (Q) value of 0.6658 and a silhouette (S) value of 0.8612. The timeline visualizes the largest 18 clusters of co-cited keywords along the horizontal timeline. Each cluster is arranged vertically in descending order of size, with nodes within the cluster arranged chronologically on the same horizontal line. Colored curves represent co-citation links added in the corresponding colored years. The closer the nodes are to the right, the more recent the topic of a cluster (1984–2024) (N=704)

In addition to keyword analysis, we further examined current trends in research on SGM tobacco use through keyword clustering. Nodes with high betweenness centrality (BC>1.0), indicating key transition points across time slices, were highlighted. In CiteSpace, such nodes are marked with purple rings, and the thickness of the ring reflects the centrality value. Figure 3 illustrates the 16 core knowledge clusters within research on SGM tobacco use. Several key clusters reveal the distinctive research architecture of the field. Cluster #0, ‘substance use’, is the largest (size = 85) and encompasses studies on multi-substance abuse patterns, particularly the combined use of alcohol, marijuana, and tobacco. Cluster #6, ‘health disparity’, addresses the health inequities related to tobacco use behaviors among sexual minorities. Cluster #7, ‘smoking cessation’, focuses on cessation interventions for sexual minorities. Cluster #9, ‘minority stress’, incorporates the theoretical framework of ‘discrimination–stress–smoking’. Cluster #10, ‘electronic cigarettes’, the most emergent cluster, highlights a growing trend in e-cigarette use among sexual minorities. Cluster #13, ‘intimate partner violence’, suggests that such experiences may be an important factor associated with increased tobacco use among sexual minorities. Cluster #14, ‘cancer care delivery’, indicates that SGM cancer patients require more support to reduce tobacco use. Cluster #16, ‘United States’, shows that much of the research on SGM tobacco use originates from US-based studies. Based on the clustering results, we classified the keywords into five main thematic categories: 1) the effects of novel tobacco products (Clusters #0 and #10); 2) subgroup differences in tobacco use (Clusters #3, #8, and #11); 3) health inequalities related to tobacco use (Clusters #1, #2, #6, and #14); 4) smoking cessation research (Clusters #5 and #7); and 5) social and psychological mechanisms, including mental health and social factors (Clusters #9 and #13).

These thematic areas reflect the broad scope of current research on SGM tobacco use and highlight emerging directions, including health inequalities, mental health, public policy, and targeted e-cigarette marketing. Collectively, these interrelated themes form a comprehensive thematic map of tobacco use trends among SGM.

## DISCUSSION

Tobacco use among sexual minorities has become a significant global public health concern. Studying trends in their tobacco use behaviors can help governments focus on this group and formulate effective policies. In terms of temporal publication trends, research on smoking among SGM has increased; however, the overall scale remains limited. For example, although there are over 100000 publications on nicotine use among adolescents, fewer than 1% focus on SGM youth. Geographically, the vast majority of studies originate from developed countries. However, the prevalence of smoking in these countries is relatively low^1^, and the health risks associated with smoking are widely recognized by their populations. In many non-Western societies, lived experiences of SGM are often deeply influenced by local traditional cultural and social norms. For example, in many parts of Asia, such as China, wherein Confucian culture remains powerful and (heterosexual) familism is often culturally and structurally prioritized, the marginality of SGMs is often further magnified by its incompatibility with traditional family culture^21^. In such cultural contexts, smoking among sexual minorities is further exacerbated by multiple social pressures. Despite these challenges, our bibliometric analysis shows that non-Western countries are significantly under-represented in research on tobacco use among sexual minorities. TheUS, which has more publications in this field than the second through fifth countries combined, serves as a model for prioritizing research on SGM smoking. Our keyword burst detection reveals that several of the earliest appearing keywords are associated with the landmark US Supreme Court case Lawrence versus Texas. This case, originating from a 1998 arrest and decided in 2003, overturned the 1986 Bowers versus Hardwick decision, marking a key advancement in the legal recognition of sexual minority rights in the US. However, recent policy developments raise significant concerns about the future of research on SGM tobacco use. In January 2025, President Trump signed the ‘Protecting Women from Gender Ideological Extremism and Restoring Biological Truth to the Federal Government’ executive order. This policy incorrectly asserts that the ‘unchanging biological reality of gender’ is determined at birth and defines sex solely based on the time of a woman’s birth. The order narrowly defines gender as either male or female, based on the ‘invariant biological reality of sex’ at birth^22^. These developments cast doubt on the future progress of research in this area.

The field has witnessed a significant shift toward interdisciplinary collaboration. While early studies were largely confined to epidemiological and clinical research in medicine and public health, recent work has expanded to include disciplines such as sociology, psychology, and economics. Scholars are increasingly using the social determinants of health framework to explore how macro-level forces – such as structural discrimination and social exclusion – affect tobacco use among SGM populations. This interdisciplinary trend has deepened in recent years, reflecting both growing global academic interest in sexual minorities and the diversification of research approaches.

Keyword co-occurrence and clustering analyses revealed five major research directions in current studies on SGM.

### The effects of novel tobacco products

Overall, SGM groups are at greater risk of tobacco use compared to cisgender heterosexual groups^23^. The introduction of new tobacco products – particularly e-cigarettes – has heightened global public health concerns^24^. Therefore, it is critical to examine differences in e-cigarette use across specific populations, especially given the targeted marketing toward sexual minorities^25^. Recent studies on e-cigarette use in SGM subgroups aim to promote better health outcomes in these populations.

### Subgroup differences in tobacco use

Adult bisexual women show the highest tobacco use risk across gender categories (37.7%), surpassing lesbian women (31.7%) and other sexual minorities^5^. According to the principle of intersectionality, multiple identities must be considered, as SGM individuals experience varying levels of tobacco use risk based on their social positions. The ‘Minorities Diminished Returns’ phenomenon – where health benefits from education are weaker among minority groups – is particularly relevant when examining education among SGMs. While higher education typically reduces tobacco use, this protective effect is significantly less pronounced for SGM groups compared to cisgender heterosexual groups^26^. Across the life course, both adolescent and older SGM individuals exhibit significantly higher risks of substance use^27,28^. Additionally, military-affiliated, minority, and rural SGM populations also demonstrate relatively high smoking rates.

### Health inequalities

Sexual minorities experience greater tobacco-related health disparities. Gay, lesbian, and bisexual smokers are more likely than cisgender heterosexual smokers to suffer from respiratory and cardiovascular diseases^29,30^. SGM cancer patients are also more likely to engage in smoking behaviors than their cisgender heterosexual counterparts^31^.

### Smoking cessation research

Most observational studies have focused on patterns of quitting behavior among SGM individuals. Specifically, older SGM cohorts are more likely to report smoking cessation compared to younger SGM women. Bisexual women also show a higher likelihood of quitting smoking than gay or lesbian women.

### Social and psychological mechanisms

A substantial body of literature indicates that violence, discrimination, stress, mental health issues, and family rejection are positively associated with tobacco use among SGM individuals^32,33^. These findings highlight the urgent need to address the unique social and psychological stressors faced by sexual minorities.

### Future perspectives

Based on the results of this systematic analysis, future research on SGM tobacco use could advance in the following areas. First, there is currently limited academic attention to developing countries such as China and India. These countries tend to have large populations of sexual minorities who use nicotine products^34^, and these individuals often face greater challenges due to cultural and religious factors^35^. We recommend that national health agencies establish dedicated funding mechanisms to support culturally-adapted research programs targeting SGM populations in these regions, while fostering international research partnerships to share best practices. Researchers are encouraged to expand studies focusing on SGM populations in these regions. Second, there is a need to deepen the understanding of nicotine use inequalities among SGM populations through an intersectionality lens. The intersectionality perspective highlights the limitations of examining health inequalities solely through single dimensions such as sexual orientation, gender, or race. The interaction between sexual orientation and social determinants – such as gender and race – is essential for comprehensively understanding nicotine use disparities and associated health inequalities among sexual minorities^5^.

Additionally, there is a relative lack of intervention and policy-oriented research on nicotine product use among sexual minorities. Most current studies emphasize influencing factors and health consequences, with comparatively little focus on intervention and policy development. We urge policymakers to implement targeted tobacco control measures, including SGM-specific cessation programs and anti-discrimination protections in healthcare settings. Future research should prioritize evidence-based interventions and policy initiatives that support smoking cessation and reduce nicotine-related health disparities among sexual minorities.

Finally, research on nicotine use among sexual minorities spans multiple disciplines, including public health, psychology, and sociology. Enhanced interdisciplinary collaboration is needed to drive theoretical innovation and practical application in research on SGM tobacco use. Academic institutions and funding bodies should create incentives for interdisciplinary research consortia to develop comprehensive approaches to SGM tobacco control.

### Limitations

This study has several limitations. First, it was limited to English-language publications indexed in the Web of Science Core Collection. It excluded non-English literature (e.g. Chinese, Spanish), non-core journals, conference papers, dissertations, and governmental reports, which may have led to the omission of important research findings. Second, most of the selected studies were observational in nature, with a notable lack of intervention-based research on smoking cessation among sexual minorities. Future studies should focus on addressing this gap. Third, complementary databases such as Scopus and PubMed were not included in this analysis, potentially limiting the comprehensiveness of the literature review. Future research will incorporate these databases for broader coverage. Finally, because bibliometric analysis focuses on representative published literature, it may overlook recent high-quality studies that have not yet accumulated citations. Continued monitoring of emerging literature is necessary.

## CONCLUSIONS

Using data mining and visualization techniques, this study constructed a comprehensive knowledge map of research on SGM tobacco use. Our findings elucidate evolving patterns and emerging trends while offering valuable perspectives to guide future investigations.

## Supplementary Material



## Data Availability

The data supporting this research are available from the authors on reasonable request.
