# Supplementary material for: Trends in global research on tobacco use among sexual and gender minorities: A bibliometric analysis, 1984–2024
Source: Tob Induc Dis. 2025 Oct 7;23:10.18332/tid/208740. doi: 10.18332/tid/208740 (PMC12505994; doi:10.18332/tid/208740)
Supplement: Supplementary file 1 [file TID-23-149-s1.pdf]

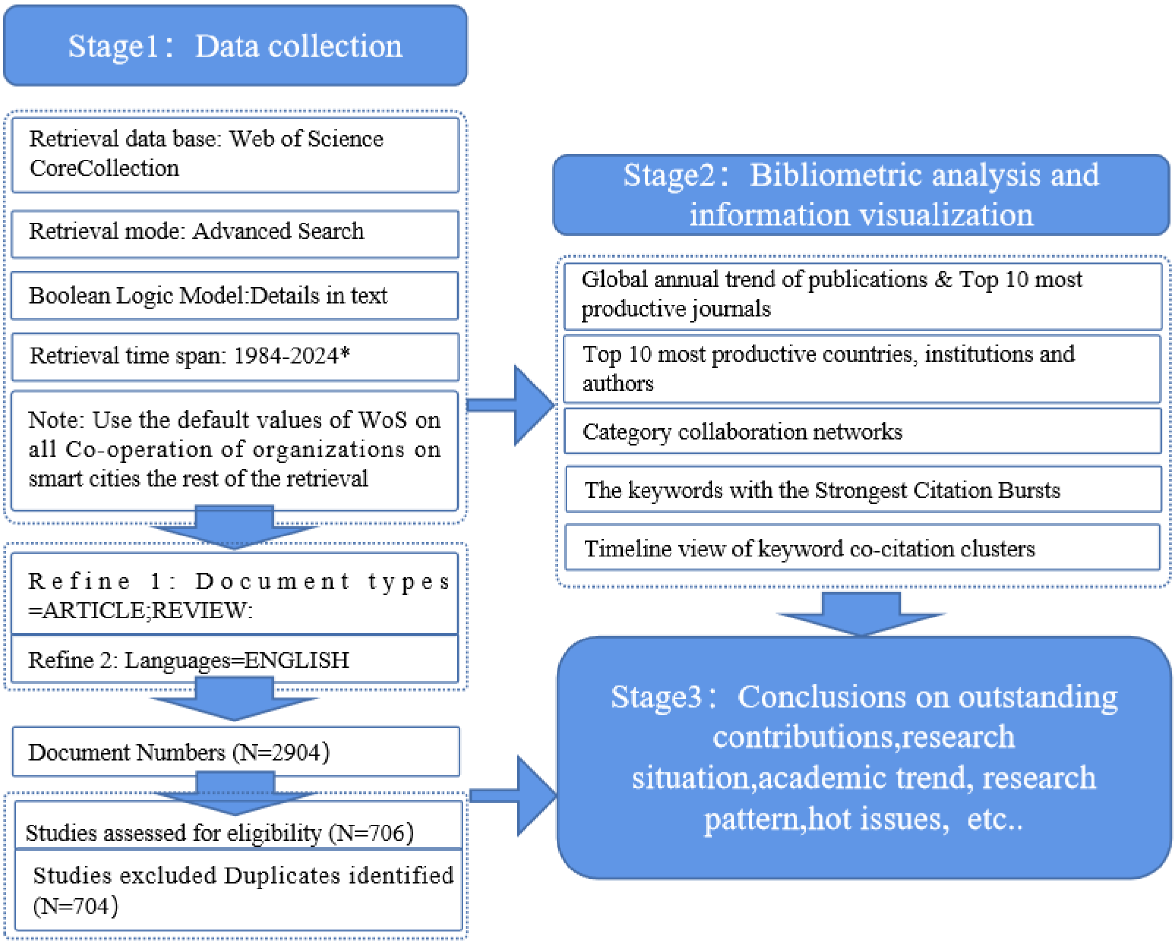

Supplementary file Figure 1. Stages of bibliometric analysis on SGM research

Supplementary file Table 1. Top 10 most productive journals (1984-2024) (N=704)

	Journal	count
TOP1	NICOTINE & TOBACCO RESEARCH	58
TOP2	LGBT HEALTH	40
TOP3	DRUG AND ALCOHOL DEPENDENCE	31
TOP4	SUBSTANCE USE & MISUSE	27
TOP5	AMERICAN JOURNAL OF PUBLIC HEALTH	27
TOP6	ADDICTIVE BEHAVIORS	24
TOP7	AMERICAN JOURNAL OF PREVENTIVE MEDICINE	22
TOP8	PREVENTIVE MEDICINE	16
TOP9	JOURNAL OF ADOLESCENT HEALTH	14
TOP10	BMC PUBLIC HEALTH	14

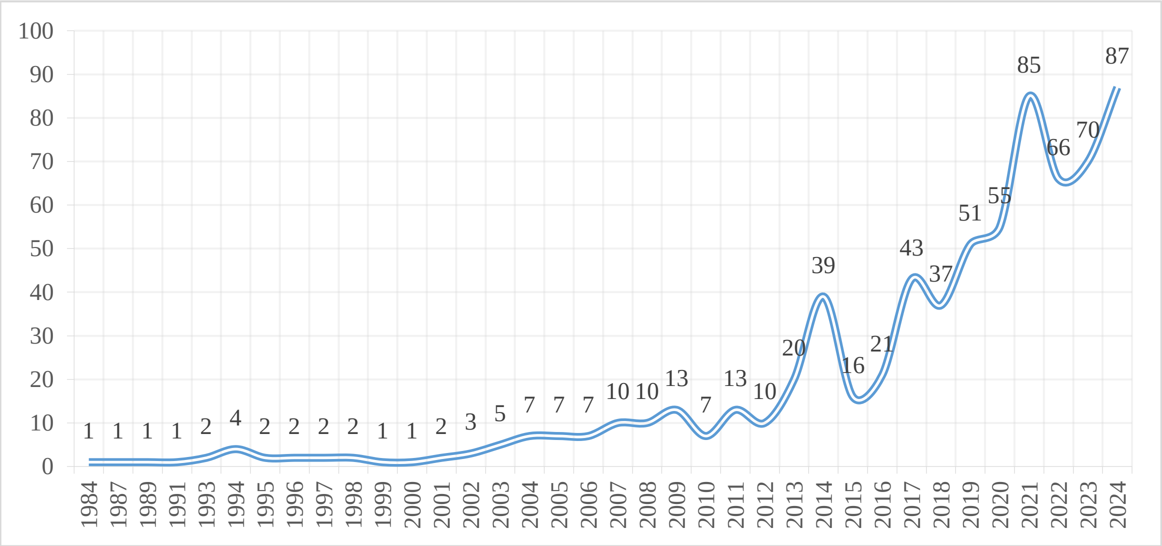

Supplementary file Figure 2. Global annual trend of publications (1984-2024) (N=704)

© 2025 Qiu L. et al
